# A Comprehensive Proteomic and Phosphoproteomic Analysis of Retinal Pigment Epithelium Reveals Multiple Pathway Alterations in Response to the Inflammatory Stimuli

**DOI:** 10.3390/ijms21093037

**Published:** 2020-04-25

**Authors:** Juha Song, Dohyun Han, Heonyi Lee, Da Jung Kim, Joo-Youn Cho, Jae-Hak Park, Seung Hyeok Seok

**Affiliations:** 1 Department of Microbiology and Immunology, and Institute of Endemic Disease, Seoul National University College of Medicine, Chongno-gu, Seoul 03080, Korea; snujuha@snu.ac.kr; 2 Department of Laboratory Animal Medicine, College of Veterinary Medicine, Seoul National University, Gwanak-gu, Seoul 08826, Korea; 3 Proteomics Core Facility, Biomedical Research Institute, Seoul National University Hospital, Seoul 03080, Korea; hdh03@snu.ac.kr (D.H.); hylee4161@gmail.com (H.L.); 4 Department of Clinical Pharmacology and Therapeutics, Seoul National University College of Medicine and Hospital, Seoul 03080, Korea; dkim3193@snu.ac.kr (D.J.K.); joocho@snu.ac.kr (J.-Y.C.); 5 Department of Biomedical Sciences, Seoul National University College of Medicine, Chongno-gu, Seoul 03080, Korea

**Keywords:** ARPE-19, inflammation, lipopolysaccharide (LPS), proteomics, phosphoproteomics, tandem-mass tags (TMTs)

## Abstract

Overwhelming and persistent inflammation of retinal pigment epithelium (RPE) induces destructive changes in the retinal environment. However, the precise mechanisms remain unclear. In this study, we aimed to investigate RPE-specific biological and metabolic responses against intense inflammation and identify the molecular characteristics determining pathological progression. We performed quantitative analyses of the proteome and phosphoproteome of the human-derived RPE cell line ARPE-19 after treatment with lipopolysaccharide (LPS) for 45 min or 24 h using the latest isobaric tandem-mass tags (TMTs) labeling approach. This approach led to the identification of 8984 proteins, of which 261 showed a 1.5-fold change in abundance after 24 h of treatment with LPS. A parallel phosphoproteome analysis identified 20,632 unique phosphopeptides from 3207 phosphoproteins with 3103 phosphorylation sites. Integrated proteomic and phosphoproteomic analyses showed significant downregulation of proteins related to mitochondrial respiration and cell cycle checkpoint, while proteins related to lipid metabolism, amino acid metabolism, cell-matrix adhesion, and endoplasmic reticulum (ER) stress were upregulated after LPS stimulation. Further, phosphorylation events in multiple pathways, including MAPKK and Wnt/β-catenin signalings, were identified as involved in LPS-triggered pathobiology. In essence, our findings reveal multiple integrated signals exerted by RPE under inflammation and are expected to give insight into the development of therapeutic interventions for RPE disorders.

## 1. Introduction

The retinal pigment epithelium (RPE) is a hexagonal pigmented cell layer located between the retina and choroid [1]. As the blood-ocular barrier component, the RPE helps maintain retinal homeostasis and visual function through mutualistic physical and metabolic interactions with the retina, and supports immune privilege by producing cell-surface and soluble inhibitory molecules such as CD86 and TGF-β [2,3,4,5]. Perturbations from aging, genetic predisposition, or environmental insults result in a wide range of retinal dystrophies [6,7]. Importantly, many studies have noted that the allostatic overload imposed during RPE pathogenesis results in inflammation [8,9,10]. Although low-grade parainflammation plays a cytoprotective role against local stress, overwhelming and persistent inflammation caused by complex biological disturbances, such as oxidative stress, complement abnormalities, and drusenoid accumulation, has been known to instigate the recruitment and activation of immune cells, exacerbating metabolic and pathobiological changes to the RPE [10]. However, the precise mechanisms causing these destructive changes to the RPE remain unclear. An improved understanding of RPE-specific inflammatory responses that determine disease progression is necessary.

Lipopolysaccharide (LPS) has been widely used to induce ocular inflammation and to recapitulate various retinal diseases such as uveitis, diabetic retinopathy (DR), and age-related macular degeneration (AMD) [11,12,13,14,15]. In the RPE, LPS is known to trigger pathological conditions such as inflammation, oxidative stress, and aging through altering multiple pathways including ERK1/2 activation, NF-κB nuclear translocation, and ROS generation [15,16]. Specifically, LPS has been reported as involved in the production of the proforms of NLRP3 inflammasome-dependent cytokines IL-1β and IL-18, whose maturation and secretion results in degeneration of the RPE [17]. Secretion of proinflammatory cytokines such as TNFα, IL-6, IL-12, IL-8, and MCP-1, which are involved in endothelial cell activation and immune cell recruitment, and tight junction disruption have also been reported in LPS-challenged RPE cells [18,19,20]. Given this, a deep understanding of the complex interplay induced by LPS and its role in pathobiology should provide valuable insights into disease mechanisms and speed development of therapeutic interventions for RPE disorders. 

Over the past decade, there has been increasing need of a systems biology approach to improve understanding of complex interplay that occurs in the RPE [21]. To date, several omics studies have sought to identify these interactions, but most studies involving the RPE have largely been limited to 2D electrophoresis (2-DE)-coupled with mass spectrometry (MS) analysis, resulting in limited protein identification and little information about functional disease states [22,23]. Recently, advances in proteome technology, and high-resolution and high-throughput mass spectrometry have enabled the intensive identification and quantification of proteomes in cells and tissues [24,25].

The high-sensitive tandem mass tag (TMT) labeling approach coupled with high-resolution mass spectrometry is recently developed proteome technique that can identify thousands of proteins with improved coverage and accuracy [26]. Here, by employing a TMT label-based quantitative analysis on the human-derived RPE cell line ARPE-19, we sought to elucidate differential expression patterns in the proteome and phosphoproteome of ARPE-19 cells exposed to LPS and unexposed control cells, and identify how aberrant pathways induce the RPE degeneration phenotype. In this study, we describe individual differentially expressed proteins (DEPs) and differentially phosphorylated proteins (DPPs) among the total network maps of ARPE-19 cells under LPS stimulation, and identify the molecular characteristics of degenerative progression.

## 2. Results

### 2.1. Induction of Inflammation on ARPE-19 Monolayer for Proteomic Analysis

**Figure 1:**
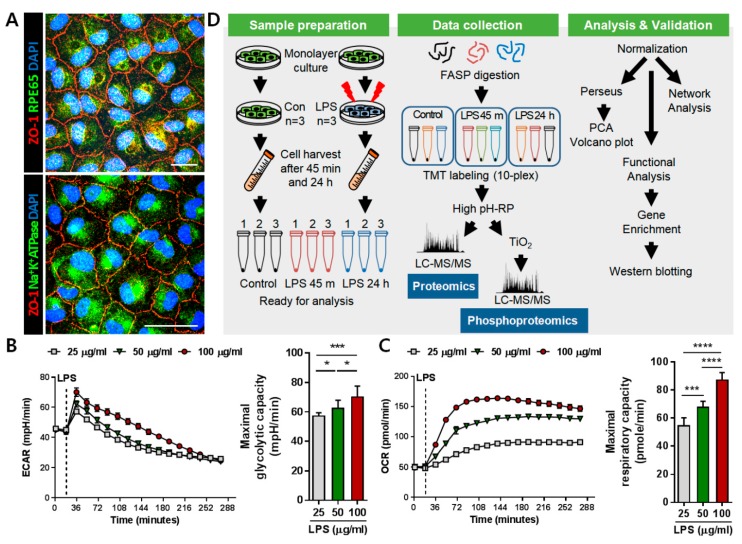
Experimental setup and workflow to investigate lipopolysaccharide (LPS)-stimulated ARPE-19 proteome and phosphoproteome. (**A**) Immunofluorescence images of polarized ARPE-19 cells stained for ZO-1 (red) and RPE65 (green) or Na^+^K^+^ATPase (green). Scale bar: 25 μm. (**B**,**C**) Real-time measurements of extracellular acidification rate (ECAR) and oxygen consumption rate (OCR) for assessing metabolic responses to different concentrations of LPS in ARPE-19 cells. Bars indicate means ± SEM. * *p* < 0.05, *** *p* < 0.001, **** *p* < 0.0001. (**D**) Pipeline for the tandem-mass tag (TMT)-based proteomic/phosphoproteomic approach to LPS-treated ARPE-19 cells.

For proteomic analysis to understand the molecular mechanisms causing pathological progression in RPE, polarized monolayer culture of ARPE-19 cells was performed. The expressions of junctional protein ZO-1, visual cycle protein RPE65, and polarity protein Na^+^K^+^ATPase were identified using immunofluorescence staining (Figure 1A). To determine the concentration of LPS that could elicit both metabolic and proinflammatory responses in RPE, extracellular acidification rate (ECAR)/oxygen consumption rate (OCR) and inflammatory cytokine levels were assessed with different concentrations of LPS. We observed that 50 μg/mL of LPS significantly increased the ECAR and OCR after treatment and facilitated the secretion of inflammatory cytokines IL-6, TNFα, IL-12, and VEGF in ARPE-19 cells, with no morphological changes but an apparent decrease in cell viability, indicating inflammation-driven pathological progression had begun within 24 h (Figure 1B,C, Figure S1). In addition, ARPE-19 cells showed significant metabolic changes between 30 min and 1 h after LPS treatment, implying that important signaling cascades has been initiated between these two time points. Therefore, we decided to investigate proteome and phosphoproteome changes in 50 μg/mL LPS-treated ARPE-19 cells to verify inflammation-driven pathology at the following time points; at 45 min for early signal transduction events and at 24 h for late proteome changes. The 10-plex TMT isobaric labeling quantitative proteomic method with high pH reversed phase (high pH-RP) chromatography was used to identify alterations in both abundance and phosphorylation status in protein extracts (Figure 1D). The data obtained from comparative MS analysis were processed with various bioinformatics tools.

### 2.2. Quantitative Proteomic and Phosphoproteomic Analyses of ARPE-19 Cells

**Figure 2:**
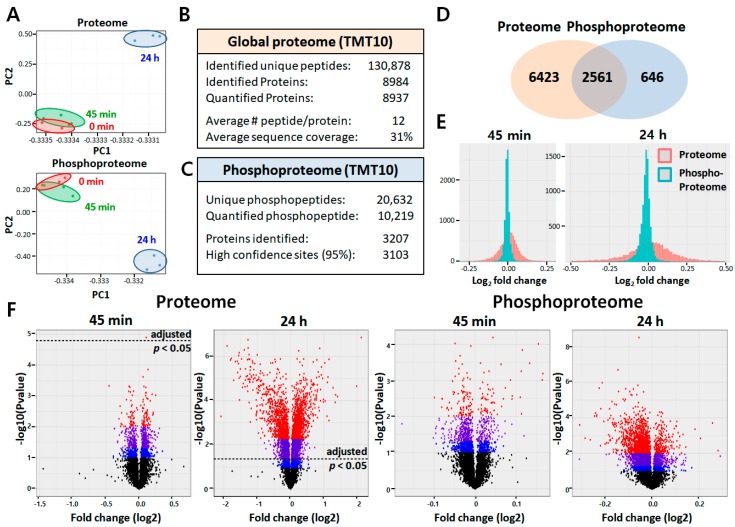
Overview of the LPS-stimulated ARPE-19 proteome and phosphoproteome. (**A**) Principal component analysis (PCA) of quantified proteins at total protein level and quantified phosphopeptides from three different treatment groups in triplicate. (**B**,**C**) Results of proteome/phosphoproteome experiments. (**D**) Venn diagram of the overlap of proteins identified in proteome profiling experiments with phosphoproteins identified in phosphoproteomic experiments. (**E**) Log fold-change distribution histogram comparing proteome (red) and phosphoproteome (blue) identified at two treatment time points. (**F**) Volcano plots from different group comparisons. Blue dots represent *p* < 0.1, |log2(fold change)| > 0, purple represent *p* < 0.05, |log2(fold change)| > 0, and red represent *p* < 0.01, |log2(fold change)| > 0. Black dotted line means *p*-value (adjusted) threshold of 0.05 to filter the statistically significant results.

The reproducibility of proteome results obtained from three biological replicates of each group was evaluated by comparing the relative protein quantification. Multiscatter plots with Pearson’s correlation coefficients between approximately 0.991 and 0.999 demonstrated strong reproducibility between biological replicates in each experimental group (Figure S2). The principle component analysis (PCA) of the data set from both the ARPE-19 proteome and phosphoproteome also showed good clustering of the biological replicates, as well as clear separation between LPS-treated and untreated control (0 min; Figure 2A). Using a false discovery rate (FDR) of 1%, we identified 130,878 unique peptides from 8984 proteins with average sequence coverage of 31% (Figure 2B, Table S1). Among the differentially expressed proteins (DEPs; Table S2), a subset of 261 proteins showed more than a 1.5-fold change in abundance after LPS challenge (adjusted *p* < 0.05; Table S3). For data sets of phosphopeptides, after internal normalization based on total amounts (Table S4), they were further normalized to the corresponding protein abundance for each phosphopeptides (Table S5). Analysis to examine the difference of phosphorylation status between control and LPS-treated ARPE-19 cells led to the identification of 20,632 unique phosphopeptides from 3207 phosphoproteins with 3103 phosphorylation sites (Figure 2C, Table S5). Among these, 618 phosphopeptides corresponding to 466 proteins, and 2774 phosphopeptides corresponding to 1358 proteins were differentially regulated after LPS challenge for 45 min and 24 h, respectively (*p* < 0.05; Table S6). In comparison of proteomic and phosphoproteomic data, we found that 2561 proteins overlapped between proteins and phosphoproteins (Figure 2D). Frequency distributions showed that most phosphoproteins were not significantly affected by LPS stimulus at either early or late time points as the average log2-fold changes centered around zero (Figure 2E). Distribution patterns of statistical significance (−log *p* values) and magnitude of change (log2 fold change) for all proteins and phosphoproteins identified at each time point were visualized using volcano plots (Figure 2F).

### 2.3. Functional Annotation of the Identified Proteins

**Figure 3:**
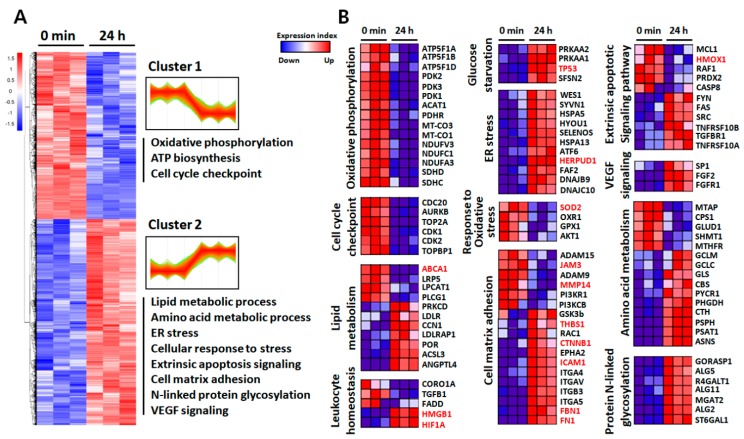
Visualization of changing proteins and their biological pathways. (**A**) Hierarchical cluster (HCL) analysis of differentially expressed proteins upon 24 h treatment of 50 μg/mL LPS, according to their abundance profile with significantly enriched biological process gene ontology (GO) terms within clusters. (**B**) Heat maps of the most enriched proteins between groups that strongly contributed to pathway enrichment scores (ES) in gene set enrichment algorithm (GSEA).

To investigate biological processes associated with inflammation-driven pathology, we performed a hierarchical clustering (HCL) analysis and gene ontology (GO) enrichment analyses. HCL analysis revealed two separate groups comprising of up- and down-regulated proteins, as compared to the untreated control (Figure 3A). The DEPs in each time point were annotated and functionally classified using the ClueGO plugin of the Cytoscape tool that can decipher GO and pathway annotation networks with a hypergeometric test and study functional correlations among pathways by the kappa coefficient calculation [27]. In contrast to the DEPs at 45 min, where few significant changes (*q* < 0.05) were observed except for KIAA1522, the 4192 DEPs at 24 h were grouped into multiple pathways (Table S7). GO analysis of the identified proteins showed strong downregulation of proteins related to mitochondrial metabolism and the cell cycle checkpoint. In contrast, perturbations in lipid and amino acid metabolism, and overall upregulation in cell–matrix adhesion, endoplasmic reticulum (ER) stress, and extrinsic apoptotic signaling were observed after LPS challenge (Figure 3A). The 261 proteins that showed more than a 1.5-fold change in abundance in response to LPS challenge also grouped multiple pathways including response to reactive oxygen species and DNA damage response (Table S3). To obtain functional protein profiles associated with the differential expression patterns, we also conducted gene set enrichment analysis (GSEA) by ranking gene products according to a differentiability statistic (e.g., ratio of expression in LPS-treated versus control) using GSEA’s Molecular Signature Database (https://www.gsea-msigdb.org/gsea/msigdb/collections.jsp; Figure 3B). 

Interestingly, with significant metabolic perturbations, two important pathways regulated by the 153 up- and downregulated proteins in our study were essential for leukocyte homeostasis and the cell–matrix adhesion. In particular, the high-mobility group box 1 (HMGB1) and hypoxia-inducible factor 1α (HIF1α), known as proangiogenic and proinflammatory factors in RPE, showed increased abundance during inflammation in the RPE. Intracellular adhesion molecule 1 (ICAM-1), fibronectin-1 (FN1), fibrillin-1 (FBN1), and thrombosponin-1 (THBS1), all associated with cellular adhesion and especially known to be increased in high-risk RPE, were also upregulated. In addition to the strong downregulation of matrix metallopeptidase 14 (MMP14), a critical enzyme for maintaining the physiological balance between synthesis and disintegration of structural elements of Bruch’s membrane (BrM), junctional adhesion molecule 3 (JAM3), which regulates the recruitment of N-cadherin and ZO-1 to tight junction formation, was also decreased by inflammation (Figure 3B).

### 2.4. Phosphoproteome Analysis

**Figure 4:**
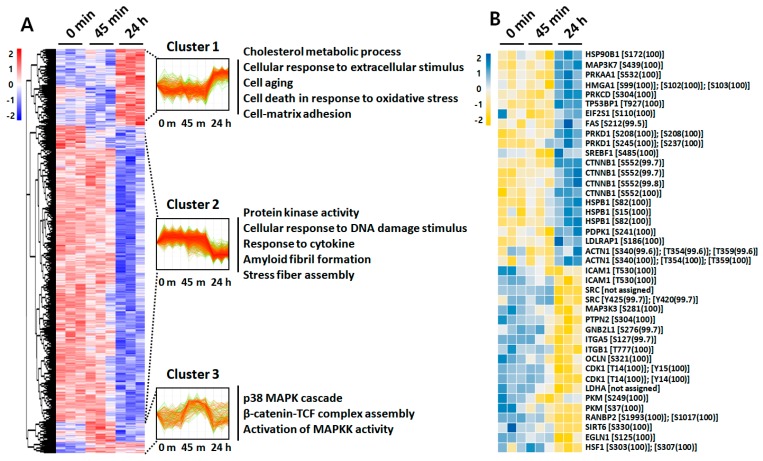
HCL analysis of the identified phosphoproteins with differential phosphorylation intensities. (**A**) HCL analysis of significantly altered phosphosites upon treatment of 50 μg/mL LPS for 45 min and 24 h, according to their abundance profile, with significantly enriched biological process GO terms within clusters. (**B**) Heat map showing phosphorylation status of 41 phosphosites that were significantly changed among three time point measurements.

To further investigate the LPS-induced signal pathways governed by protein phosphorylation and dephosphorylation, we first performed HCL analysis using a phosphoproteome data set. Interestingly, clustering of the phosphoproteome revealed a clear segregation into three clusters, reflecting three distinct patterns of phosphorylation progression by LPS challenging (Figure 4A). Differentially phosphorylated peptides (DPPs) of cluster 1 (449 of 2376, 18.90%), which exhibited increased phosphorylation at 24 h post-challenge, were selectively enriched for several GO terms, including cholesterol metabolic process, cell aging, cell–matrix adhesion, and cell death. DPPs of cluster 2 (1742 of 2376, 73.32%), which exhibited dephosphorylation at 24 h post-challenge, were enriched for GO terms including protein kinase activity, cellular response to DNA damage, and response to cytokine. Finally, DPPs of cluster 3 (185 of 2376, 7.79%) that exhibited phosphorylation at 45 min after LPS challenge were enriched for GO terms including the p38 MAPK cascade and β-catenin-TCF complex assembly (Figure 4A, Table S8). For detailed identification of early phosphorylation events after LPS challenge, we reperformed HCL analysis using phosphoproteome data sets of 0 min and 45 min (Figure S3A). Among the two distinct clusters, DPPs of cluster 1 (196 of 618, 31.88%) that exhibited phosphorylation at 45 min after LPS challenge were also enriched for I-κB phosphorylation and cell death, whereas cluster 2 (422 of 618, 68.45%) was enriched for GO terms including ER stress and regulation of DNA repair. The significantly altered phosphorylation status of key protein components after stimulation with LPS for 45 min and 24 h was visualized using a heatmap with identified phosphosites (Figure 4B, Table S9).

Interestingly, protein kinase C delta (PRKCD) involved in NF-κB activation was phosphorylated at residue Ser-304 (100%). In addition, β-catenin (CTNNB1), involved in the regulation of inflammation via crosstalk with NF-κB, was phosphorylated at residue Ser-552 (99.8%) after LPS challenge for 24 h, whereas occludin (OCLN), whose phosphorylation has been known to occur in the maintenance of tight junction integrity, was identified to be significantly dephosphorylated at residue Ser-321 (100%). The significantly altered phosphorylation status of key protein components by stimulation with LPS for 45 min was also identified with phosphosites in Figure S3B.

### 2.5. Protein Interaction Network of LPS-Treated ARPE-19 Cells

**Figure 5:**
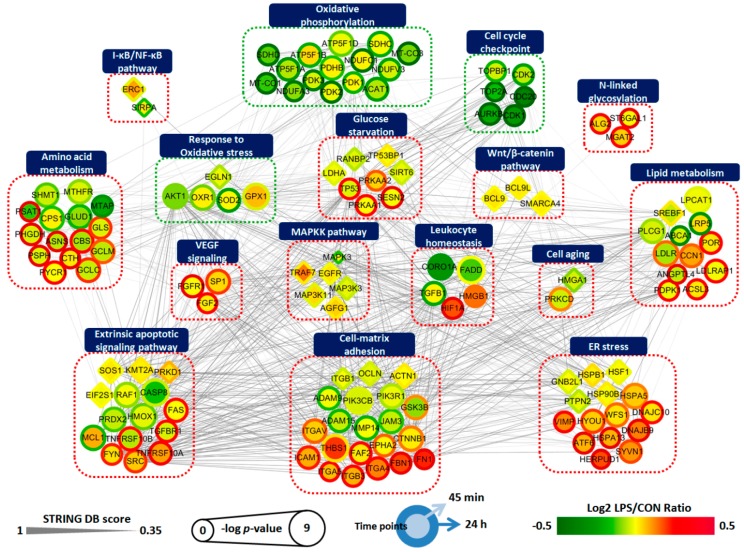
Network analysis of proteins related to inflammation-driven pathology of ARPE-19 cells. A total of 136 differentially expressed proteins and phosphoproteins involved in differentially regulated biological processes were grouped according to their function. Circle indicates DEP and diamond indicates DPP. Node colors represent an increase (red) and decrease (green) in LPS-treated ARPE-19 cells for 45 min (center) and 24 h (boundary) compared to untreated controls. The color bar represents the gradient of log2 protein ratios. The size of a node represents –log10(*p*-value). The edges represent PPIs obtained from the STRING database [28]. Pathways with red dotted lines indicate clusters upregulated by LPS and pathways with green dotted lines indicate clusters downregulated by LPS. All are indicated by gene symbol.

To examine the relationship between the significantly altered proteome and phosphoproteome, we constructed a protein–protein interaction (PPI) network using Cytoscape software. The separated analysis of the proteomics and phosphoproteomics datasets were depicted in one-dimensional views of cellular processes by integrating the information regarding protein abundances, activation status, and molecular interactions, with the goal of determining their complex interplay during inflammation-driven pathogenesis. In this network modeling, we focused on the 106 DEPs and 30 DPPs that were selected based on the GSEA and ClueGO database (Figure 3B, Figure 4B, Figure S3B). As shown in Figure 5, a total of 136 nodes were largely interconnected with 1094 edges, suggesting they are multifunctional and interdependent. These nodes were grouped and represented by 16 GO-biological process terms that were closely associated with RPE pathobiology. 

Finally, to validate our MS-based quantification of protein and phosphoprotein, we performed Western blot analysis for the two proteins (ICAM-1 and heme oxygenase-1, HMOX-1) and one phosphoprotein (β-catenin) that were involved in distinct pathways. Consistent with the proteome data, ICAM-1 was shown to be upregulated, while HMOX-1 was downregulated after LPS treatment for 24 h (Figure S4A). The phosphorylation of β-catenin at Ser-552 residue was also identified as increased by LPS challenge for 24 h (Figure S4B). These findings indicate that the TMT labeling-based proteomic and phosphoproteomic analysis performed in this study possesses sufficient analytical power to confidently reveal changes in protein and phosphoprotein levels relevant to inflammation-driven pathology.

## 3. Discussion

In this study, we aimed to create a global overview of inflammation-driven biological and metabolic alterations in RPE that causes destructive changes in the retinal environment by using the latest tag based multiplex proteomic/phosphoproteomic quantitative analysis. Here, a total of 4490 differentially expressed proteins and 1561 differentially phosphorylated proteins were identified in LPS-treated ARPE-19 cells (Tables S2 and S6). We revealed significant downregulation of proteins related to mitochondrial respiration, cell cycle checkpoint, and antioxidant system, which were identified as interconnected with robust perturbations in lipid metabolism, amino acid metabolism, ER stress, cell–matrix interaction, and apoptosis. Early phosphorylation events associated with the MAPKK pathway, the Wnt/β-catenin pathway, and I-κB/NF-κB signaling, and subsequent phosphorylation/dephosphorylation of proteins related to cell aging, and cell–matrix adhesion were identified as involved in pathogenesis. To our knowledge, this is the first large-scale proteome/phosphoproteome analysis of inflammation-triggered RPE cells, providing a comprehensive picture of the dynamically intertwined protein interaction network.

Understanding the pathological mechanisms that damage the RPE during aging and disease has been challenging because of the lack of appropriate models that can faithfully recapitulate its multifactorial and complex pathobiology [21]. Hence, integrated understanding of multiple models was recommended [21]. LPS, an agonist of toll-like receptor 4 (TLR4) used in this study for inducing inflammatory pathology, has frequently been utilized in RPE studies as a priming or stimulating agent because LPS-triggered downstream signals and phenotypes have been reported as similar to those seen in RPE pathobiology [15,16,17,18,19,20], as mentioned in the introduction. Interestingly, our results identified an early and strong metabolic response after LPS challenge (Figure 1B,C), implying that subsequent metabolic overload of the mitochondria could occur, leading to various biological alterations. Indeed, after 24 h after treatment with LPS, robustly interconnected perturbations in ER stress, cell–matrix adhesion, antioxidant mechanism, and apoptosis were observed (Figure 5).

Our study presents deep profiling of inflammation-driven phosphorylation events in RPE cells. At 45 min after LPS stimulation, phosphorylation events including SOS1 on Ser-1134, TRAF7 on Ser-61, MAP3K7 on Ser-389, and MAP3K11 on Thr-752 were identified (Figure S3B), implying the subsequent activation of MAPK and NF-κB signaling. In addition, proteins involved in the Wnt/β-catenin pathway, such as BCL9 and SMARCA4, were also phosphorylated. These interconnected signaling pathways are implicated in a variety of signaling cascades wherein various extracellular stresses induce inflammation. Activation of MAPK and NF-κB pathway and their involvement in the secretion of inflammatory cytokine such as IL-6 and IL-8 has been reported previously in RPE cells after LPS stimulation [29,30]. Increased phosphorylation of ERK 1/2 was also identified in inflamed and degenerative RPE during the pathogenesis of AMD and has been suggested as a potent therapeutic target [31]. Furthermore, the pathogenic role of Wnt/β-catenin signaling, via crosstalking with many other signaling pathways including NF-κB and HIF1α, has been demonstrated in RPE-oriented degenerative disorders [32,33,34]. Considering our proteome and phosphoproteome data showed increased β-catenin expression along with elevated phosphorylation at residue Ser-552 upon LPS stimulation for 24 h (Figure 4B), Wnt/β-catenin signaling appears to also be activated in our inflammation-induced RPE, whereas expression of PI3K/Akt, known to phosphorylate β-catenin in a Wnt-independent fashion [35], was downregulated (Figure 4B). Instead, PRKAA2 (known as AMPKα2) expression, another protein known to phosphorylate β-catenin [36], was upregulated (Figure 4B), suggesting a possible crosstalk between AMPK and the Wnt/β-catenin signaling pathway in inflammation-induced RPE. Phosphorylation of β-catenin on Ser-552 and its involvement in activation of aerobic glycolysis for production of angiogenic factors have been previously described in various diseases such as cancer and AMD [37,38,39]. A similar β-catenin phosphorylation response was observed during an oxidative stress-induced detrimental cellular dedifferentiation process in mouse RPE [40]. In addition, the activation of the Wnt/β-catenin pathway is known to induce VEGF, TNFα, and ICAM-1 in ARPE-19 cells [41].

Here, in response to inflammatory stimuli, prominent perturbations in mitochondria, lipid, and amino acid metabolic pathways were identified in RPE. Disturbances to metabolic stability have been implicated in inflammation and degenerative progression [42]. Of note, mitochondria in the RPE have been reported as a primary site of pathology, especially with aging [43]. Growing evidence has highlighted inflammation/oxidative stress-induced mitochondrial dysfunction and subsequent metabolic shifts from oxidative phosphorylation (OXPHOS) to glycolysis in RPE, thereby reducing the flow of glucose to photoreceptors, a process implicated in retinal degeneration [43,44,45]. In accordance with these previous studies, our results also showed strongly suppressed mitochondrial function with reduced OXPHOS-related proteins such as ATP synthase (ATP5F1A-D), mitochondrially encoded cytochrome c oxidase (MT-CO1-3), and pyruvate dehydrogenase kinase (PDK1-3). Given our results showing an LPS-induced respiratory burst (Figure 1C) and substantial reduction of the mitochondrial antioxidant enzyme SOD2 (Figure 3B), the mitochondrial dysfunction is thought to be due to the elevated ROS level within the mitochondria. SOD2 reduction and mitochondrial dysfunction have been reported in mouse under light damages [46]. A positive correlation between SOD2 deficiency and the development of retinal degeneration has also been demonstrated previously [47,48]. Although the molecular mechanism of inflammation-induced SOD2 reduction remains unknown, activated p53 signaling (Figure 3B) is suspected to be involved [49].

Aberrant lipid accumulation occurs in both the RPE and BrM during aging and disease due to disturbances in lipid metabolism, which contributes to deleterious consequences on RPE and retinal function [50]. In particular, genetic mutations or reduced function in proteins associated with lipid trafficking, such as LDLR, ABCG1, and ABCA1, have been known to induce deposition of lipid-rich particles within the RPE and BrM as well as inflammation and degeneration in both RPE and retina [51,52]. Of note, ABCA1 has been reported as critical for maintaining lipid homeostasis and survival of RPE [53]. ABCA1, which acts as an anti-inflammatory receptor by suppressing the expression of inflammatory factors, has previously been demonstrated as blocked by the inflammatory stimuli such as COX-2 and LPS in macrophages [54]. Similarly, our current proteomic analyses also showed a strong downregulation of ABCA1 with corresponding upregulation of LDLR (Figure 3B), implying decreased cholesterol efflux with increased LDL cholesterol uptake in RPE. ROS- and NF-κB-dependent pathways are suspected as possible underlying mechanisms for inflammation-driven ABCA1 downregulation [55].

We also showed significant alterations in amino acid metabolism during inflammation. In particular, the expression of enzymes involved in folic acid and derivative biosynthetic processes, such as methylenetetrahydrofolate dehydrogenase (MTHFD1), methylene tetrahydrofolate reductase (MTHFR), and methylthioadenosine phosphorylase (MTAP), was significantly downregulated upon LPS stimulation (Figure 3B), implying an overall increase of homocysteine levels. Although cystathionine β-synthase (CBS) and cystathionine-γ-lyase (CTH), enzymes involved in catalyzing homocysteine to glutathione, were simultaneously increased, our results revealed an increased abundance of homocysteine-responsive ER-resident protein HERPUD1 (Figure 3B), suggesting that elevated homocysteine levels had already triggered ER stress in RPE. While homocysteine has been reported as strong inducer of inflammation, oxidative stress, and blood-retinal barrier (BRB) disruption [56,57], HERPUD1 has also been known to elevate the level of amyloid-β, which exacerbates the drusen deposition underlying the RPE layer [58]. Indeed, hyperhomocysteinemia due to a lack of CBS has been associated with several human visual disorders, such as DR and AMD [59,60].

ARPE-19 cells, as compared to human primary RPE, has been reported to have several limitations, such as a lack of major RPE differentiation and polarity markers with low transepithelial electrical resistance (TEER) [61]. Nevertheless, ARPE-19 is a good alternative model for human primary RPE having the obvious difficulties in obtaining human eye tissues and the inconsistency due to individual heterogeneity. In view of this, our results obtained from a very simplified model, which used ‘standard’ RPE cell lines treated with a simple but widely known inflammatory stimulus are expected to function as a framework for future mechanistic studies, providing a basis for generalizable information relevant to inflammatory responses in the RPE. To further confirm the specific pathways involved in RPE pathobiology, the use of induced pluripotent stem cell (iPS)-derived RPE from patients, which has been proven beneficial at uncovering the role of specific genes or proteins during pathogenesis, might be helpful.

Collectively, we adopted the latest proteome and phosphoproteome profiling approach and applied it to the study of the pathobiology of RPE in response to overwhelming inflammation. We presented various concerted and integrated protein signals involved in the biological and metabolic responses of inflammation-triggered ARPE-19 cells. By layering the proteomic network tightly interwoven into pathogenesis, we identified numerous protein and phosphoprotein signatures possessing functional importance in RPE. Our descriptive and detailed findings could provide a steppingstone toward improved understanding of the RPE-specific pathologic responses against intense inflammation that might occur during aging and disease, and should be followed by mechanistic studies. Our data are expected to have profound implications for understanding disease mechanisms and for developing therapeutic intervention for RPE disorders.

## 4. Materials and Methods

### 4.1. Cell Line Culture

Human ARPE-19 cells were purchased from the ATCC (CRL-2302, Manassas, VA, USA) and cultured in Dulbecco’s modified essential medium and Ham’s F12 (DMEM/F12, 1:1, Gibco, Carlsbad, CA, USA) supplemented with 10% fetal bovine serum (FBS, Gibco), 2 mM L-glutamine (Gibco), and 1% penicillin-streptomycin (Gibco) at 37 °C under 5% CO_2_ in a humidified incubator. For confluent ARPE-19 monolayer culture, cells were seeded onto a 100-mm dish tissue culture plate (3.5 × 10^6^ cells per well; Corning Glass Works, Corning, NY, USA) and differentiated in DMEM/F12 media with reduced serum (1% FBS) for 7 days. The culture medium was changed every 2 days. Polarized monolayer cultures showing TEER greater than 40 Ωcm^2^ were used for the downstream experiments.

### 4.2. Immunofluorescence Staining

After fixed with 4% paraformaldehyde for 15 min at room temperature, ARPE-19 cells were washed with PBS containing 0.3% Triton X-100 (PBST) three times and blocked at 37 °C for 1 h in PBST supplemented with 5% FBS (Gibco). Then, the cells were incubated with the following primary antibodies at 4 °C overnight: rabbit anti-ZO-1 (Santa Cruz Biotechnology Inc., Santa Cruz, CA, USA), mouse anti-Na^+^K^+^ATPase (Santa Cruz Biotechnology Inc.), and mouse anti-RPE-65 (Novus Biologicals, Littleton, CO, USA). Cells were washed 3 times for 5 min with PBST and incubated with Alexa Fluor 488-labeled goat anti-rabbit IgG (Molecular Probes, Eugene, OR, USA) and Alexa Fluor 635-labeled goat anti-mouse IgG (Molecular Probes) for 1 h at room temperature. Stained cells were examined under Olympus FV1000 Confocal Scanning Scope (Olympus, Tokyo, Japan).

### 4.3. LPS Treatment and Sample Preparation

ARPE-19 cells were stimulated with different concentrations of LPS (from *Salmonella enteritidis*, Sigma, St. Louis, MO, USA) for the indicated periods (45 min and 24 h; *n* = 3). Unchallenged cells (denoted as 0 min) were used as controls. Then, LPS-treated and control ARPE-19 cells were immediately washed twice and harvested with 1 mL of ice-cold PBS containing protease inhibitor and phosphatase inhibitor (GenDepot, Barker, TX, USA). Cells were stored at −80 °C for the downstream proteomic and phosphoproteomic analysis.

### 4.4. Protein Digestion

Cell lysates were digested using the Filter Aided Sample Preparation (FASP) method [62,63]. First, cell pellets were lysed with the lysis buffer (4% SDS, 1 mM DTT, and 0.1 M HEPES pH 7.5). Protein concentration was determined using the BCA reducing compatible kit (Thermo Fisher Scientific, Rockford, IL, USA). After acetone precipitation with 200 μg of protein, pellets were resolved with the SDT buffer (4% SDS, 2 mM TCEP, and 10 mM CAA in 0.1 M HEPES, pH 7.5) and heated on a heat block at 95 °C for 15 min. Samples were then mixed with 200 μL of UA solution (8 M UREA in 0.1 M HEPES, pH 7.5). Next, the samples were subjected to a 30 kDa Amicon Ultracel filter (Millipore, Burlington, MA, USA) and centrifuged at 14,000× *g* for 15 min. After two buffer exchanges with UA solution, further buffer exchanges were performed with 40 mM of HEPES pH 7.5 three times. Protein digestion was performed overnight at 37 °C using a trypsin/LysC mixture at a 100:1 protein to enzyme ratio (*w*/*w*). Peptides were collected by centrifugation.

### 4.5. TMT 10-Plex Labeling

Before TMT labeling, the peptide concentration of each sample was measured using a tryptophan assay [64]. TMT 10-plex labeling was then performed with some modifications to the manufacturer’s protocol [62]. Peptides were labeled with TMT 10-plex reagents, such that peptides from control replicates were conjugated to tags 126, 127N, and 127C, LPS treated (45 min) replicates with tags 128N, 128C, and 129N, and LPS treated (24 h) replicates with tags 129C, 130N, and 130C, then pooled sample with tag 131. The TMT reagent that dissolved in 100% of acetonitrile (ACN) was added to 300 μg of peptide along with ACN to give a final concentration of 30% *v*/*v*. After incubation at room temperature for 1 h, the TMT-labeled samples were pooled at a 1:1:1:1:1:1:1:1:1:1 ratio. The pooled sample was dried almost completely and desalted using a C18 solid-phase extraction (SPE) column (Waters).

### 4.6. High-pH Peptide Fractionation

The desalted pooled peptides were fractionated using an Agilent 1260 bioinert HPLC (Agilent, Santa Clara, CA, USA) equipped with an analytical column (4.6 mm × 250 mm, 5 μm particle) as described previously [62,65]. High-pH peptide fractionation was performed with a 50 min gradient of 5–35% Solvent B at a flow rate of 0.2 mL·min^−1^ using Solvent A (15 mm ammonium hydroxide) and Solvent B (15 mm ammonium hydroxide in 90% ACN). Totally, 96 fractions were collected every minute from 1 to 50 min. Subsequently, 96 fractions were non-contiguously pooled into 24 fractions. From each pooled fraction, we collected 5% of the fraction into an HPLC vial for global proteome analysis by LC–MS/MS [26]. For enrichment of phosphopeptide, the remaining 95% of each fraction were combined into 12 fractions.

### 4.7. Phosphopeptide Enrichment Using TiO_2_

Phosphopeptide enrichment was carried out as described in Humphrey SJ, et al. 2018 [66] with some modifications. Briefly, dried peptide fractions were resuspended using loading buffer composed of 6% (*v*/*v*) TFA and 80% ACN. TiO2 beads (Titansphere, GL Sciences Inc., Tokyo, Japan) were subsequently added to the peptides at a ratio of 10:1 beads/protein and incubated in a thermomixer at 2000 rpm for 30 min at 40 °C. After centrifugation for 1 min at 3500× *g*, the supernatant (containing non-phosphopeptides) was aspirated and discarded. Beads were suspended in the wash buffer (60% ACN and 1% TFA). Washes were performed by a centrifugation four times with 1 mL of the wash buffer. Beads that were suspended in 100 μL of the transfer buffer (60% ACN and 0.1% TFA) were transferred to the top of a C8 StageTip, and centrifuged for 3–5 min at 3000 rpm. Phosphopeptides were eluted two times with 30 μL of the elution buffer (40% ACN and 15% ammonia solution) and collected by centrifugation into clean PCR tubes. Eluents were dried in a SpeedVac and acidified by the addition of 10% TFA. Phosphopeptides were desalted by loading onto a C18-SDB-RPS stageTip as described previously [67]. StageTips were washed with 0.2% TFA, and phosphopeptides were eluted with 60 μL of elution buffer (80% ACN and 5% NH_4_OH). Samples were immediately concentrated in a SpeedVac for 30 min at 45 °C. Desalted peptides were resuspended in LC injection buffer (2% ACN and 0.1% formic acid) for LC–MS/MS analysis.

### 4.8. Mass Spectrometric Analysis and Database Search

Peptide samples were analyzed using a Q-Exactive plus mass spectrometer (Thermo Fisher Scientific, Bremen, Germany) coupled with an Easy-nLC system (Thermo Fisher Scientific, Odense, Denmark) as described previously [65]. Peptides were resuspended in 2% ACN and 0.1% formic acid and separated on a two-column system with a trap column and an analytical column (EASY-Spray column, 2 m particle size, 100 Å pore size, 75 μm id × 50 cm length, Thermo Fisher Scientific). Peptides were separated with a linear gradient of 8–32% solvent B (80% ACN and 0.1% formic acid) over 180 min at a flow rate of 300 nL/min. Spray voltage was set to 2.1 kV. Full MS was acquired with a mass range of 350–1800 m/z at a resolution of 70,000 at m/z 200. MS2 scans were acquired at a resolution of 35,000. The top 15 precursor mass were selected in a data dependent acquisition (DDA) mode for MS/MS fragmentation by higher-energy collisional dissociation (HCD) at 32% normalized collision energy (NCE). The dynamic exclusion duration was set at 40 s and the isolation width was 0.7 Th with no offset. The resulting MS/MS spectra were searched against the UNIPROT human protein database (Uniprot release 2014_12, 88,657 entries) with the SEQUEST-HT algorithm in Proteome Discoverer 2.2 (Thermo Fisher Scientific, Bremen, Germany). A maximum of two missed cleavage sites was allowed. Mass tolerances for precursor ions and fragment ions were set to 20 ppm and 0.02 Da, respectively. Carbamidomethylation of cysteine residues (+57.021 Da) and TMT tags on lysine residues and peptide N-termini (+229.163 Da) were set as fixed modification. Oxidation of methionine residues (+15.995 Da) and phosphorylation of serine, threonine, and tyrosine residues (+79.966 Da) were set as variable modifications. The false discovery rate (FDR) was determined using the percolator node. The FDR was set to 0.01 at the peptide spectral match (PSM), peptide, and protein levels. Only the PSMs that contained all ten reporter ions were considered for quantification. Proteins were quantified by summing reporter ion intensities across all matching PSMs. Normalization was performed based on the total reporter ion intensity in each channel. The mass spectrometry proteomics data have been deposited to the ProteomeXchange Consortium via the PRIDE [68] partner repository with the dataset identifier PXD018257 (proteome) and PXD018260 (phosphoproteome).

### 4.9. Bioinformatics Analysis

Statistical analysis for proteomic data was performed using the Perseus software [69]. Student’s *t* test was employed for the proteome data set to identify statistically significant (permutation-based FDR at 5%) differences in expression at the protein levels, and for the phosphoproteome data set to identify statistically significant (*p* < 0.05) differences at the phosphorylation levels. For the hierarchical clustering analysis, multiple sample tests using one-way ANOVA was conducted (*p* < 0.05). Gene ontology (GO) annotation and pathway enrichment analysis were implemented with ClueGO plugin [70] of Cytoscape tool. Protein quantification data were also analyzed using gene set enrichment analysis (GSEA), and shown with density plots depicting the abundance of a gene set relative to the entire dataset (https://www.gsea-msigdb.org/gsea/msigdb/collections.jsp). Protein interaction network analyses of the significantly altered proteome and phosphoproteome in RPE data sets were performed with Cytoscape (v3.7.1) software [71] using the STRING database [28].

### 4.10. ECAR and OCR Measurements

The ECAR (in mpH/min) and OCR (in pmol/min) were measured using the Seahorse XF-24 metabolic extracellular flux analyzer (Seahorse Bioscience, Billerica, MA, USA) [72]. ARPE-19 cells were seeded at a density of 5 × 10^4^ cells per well in Seahorse cell plates and differentiated for 7 days. On the day of analysis, cells were washed two times with glucose free assay media (Seahorse Bioscience), and the ECAR and OCR in response to different concentrations (25, 50, and 100 μg/mL) of LPS were assessed in glucose-containing assay media. Experiments with the Seahorse system were performed according to the assay protocol consisted of repeated cycles of 2 min mixture; 2 min wait; and 4–5 min measurement. The ECAR and OCR were recorded and then calculated.

### 4.11. Cell Viability Assay

ARPE-19 cells were seeded 100 μL in a 96-well plate at a density of 2.5 × 10^4^ cells per well, and maintained in DMEM/F12 media with reduced serum (1% FBS) for 7 days. The culture medium was changes every 2 days. The cells were stimulated with various concentrations of LPS for 24 h, and maintained in a humidified incubator at 37 °C in 5% CO_2_. On the day of analysis, 10 μL of 5 mg/mL 3-(4, 5-Dimethylthiazol-2-yl)-2,5-diphenyltetrazolium bromide (MTT, Sigma) was added to each well and incubated for an additional 4 h. After being centrifuged at 2400 rpm for 5 min, the supernatant was aspirated carefully. Then, 100 μL dimethylsulfoxide (DMSO) was added to each well and incubated for 20 min to dissolve the formazan crystals. The absorbance was recorded at 560 nm with a microculture plate reader (Becton-Dickinson Labware, Lincoln Park, NJ, USA).

### 4.12. ELISA

ARPE-19 cells were treated with LPS for 24 h. The media was collected, centrifuged at 2500 rpm for 5 min to remove particulates, and stored at −80 °C until ELISA was performed. The protein concentration of the cells in each well was measured using bicinchoninic acid (BCA) protein assay kit (Thermo, Waltham, MA, USA) for the normalization of downstream cytokine quantification. Cytokine production from LPS-treated ARPE-19 cells was measured using the Duoset ELISA kit (R&D System, Minneapolis, MN, USA) according to the manufacturer’s instructions.

### 4.13. Western Blotting

The cells were harvested with the 1X sample buffer (Biosesang, Seongnam, GG, Korea) containing a protease inhibitor and phosphatase inhibitor cocktail (GenDepot). Then, the total cell lysates were sonicated for 30 s and heated to 100 °C for 10 min. The proteins were separated on 8% polyacrylamide gels followed by transfer onto polyvinylidene difluoride (PVDF) membranes (Merck Millipore, Burlington, MA, USA). ICAM-1 (Santa Cruz Biotechnology Inc.), HMOX-1 (Santa Cruz Biotechnology Inc.), phospho-β-catenin (Ser552; Cell Signaling Technology, Danvers, MA, USA), β-catenin (Santa Cruz Biotechnology Inc.), and β-actin (Santa Cruz Biotechnology Inc.) were used at 1/2000 dilutions.

### 4.14. Statistical Analysis

All results are expressed as the means ± SEM and visualized using Prism version 6 (Graphpad Software, La Jolla, CA, USA). The student’s unpaired *t* test (two-tailed) was used to calculate statistical significance between experimental groups. *p* < 0.05 was defined to be statistically significant.

## 5. Conclusions

Inflammation beyond homeostasis-maintaining parainflammation in the RPE is detrimental, and strongly implicated in age-related ocular pathogenesis. To gain insight into the molecular mechanisms underlying inflammation-driven pathological changes, we employed a systems biology approach using ARPE-19 cells stimulated with LPS to elicit metabolic and inflammatory response. The proteome and phosphoproteome results obtained here shed light on the unique protein signatures altered and activated by inflammation in RPE. In essence, we highlighted relationships between metabolic alterations and pathobiological changes in response to the inflammatory stimuli, and identified four major patterns: (1) activation of early signaling cascades including MAPK, NF-κB, and the Wnt/β-catenin pathway, (2) strong downregulation of mitochondrial metabolism and cell cycle checkpoint, (3) perturbations in lipid and amino acid metabolism, and (4) upregulation of cell-matrix adhesion, ER stress, and apoptosis pathways.

## Supplementary Materials

Supplementary materials can be found at https://www.mdpi.com/1422-0067/21/9/3037/s1. Figure S1. Cytokine production of LPS-treated APRE-19 cells. Figure S2. Reproducibility of global proteomic quantification. Figure S3. Hierarchical clustering of the identified phosphoproteins with differential phosphosite intensities. Figure S4. Western blot analysis of proteins and phosphoprotein included in the pathways related to inflammation-driven pathogenesis in ARPE-19 cells. Table S1. List of the identified protein in global proteomic analysis. Table S2. List of differentially expressed proteins (DEPs; *q* value < 0.05). Table S3. A subset of proteins and pathways which showed more than a 1.5-fold change in abundance after LPS challenge for 24 h (adjusted *p* value < 0.05). Table S4. List of the identified phosphopeptides. Table S5. List of the quantified phosphopeptides. Table S6. List of differentially phosphorylated proteins (DPPs; *p* value < 0.05). Table S7. Enriched GO biological process pathways in protein abundances of LPS-treated ARPE-19 cells compared to untreated controls (FDR < 0.05). Table S8. Enriched GO biological process pathways within each cluster of LPS-stimulated ARPE-19 phosphoproteome (one-way ANOVA, *p* < 0.05). Table S9. Enriched GO biological process pathways within each cluster of LPS-stimulated ARPE-19 phosphoproteome (Student’s *t* test, *p* < 0.05).
