# Supplementary material for: A Comprehensive Proteomic and Phosphoproteomic Analysis of Retinal Pigment Epithelium Reveals Multiple Pathway Alterations in Response to the Inflammatory Stimuli
Source: Int J Mol Sci. 2020 Apr 25;21(9):3037. doi: 10.3390/ijms21093037 (PMC7246457; doi:10.3390/ijms21093037)
Supplement: Supplementary file 1 [file ijms-21-03037-s001.zip › Supplementary Figures.pdf]

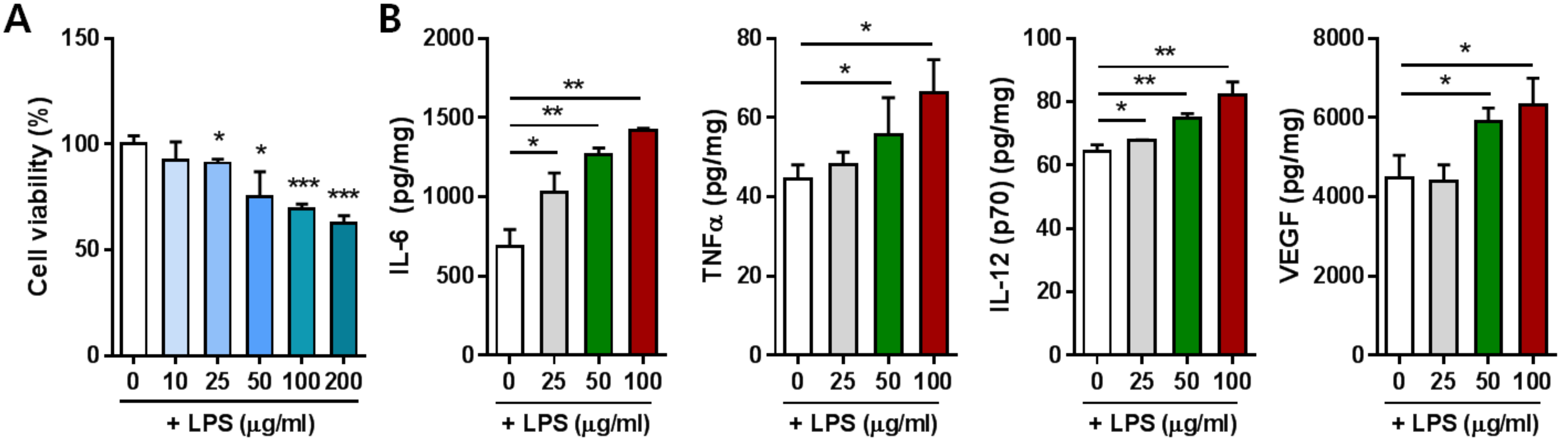


**Figure S1.** Cytokine production of LPS-treated APRE-19 cells. (A) Cell viability measurement of ARPE-19 cells stimulated with different concentrations of LPS for 24 h. (B) Cytokine production of ARPE-19 cells treated with different concentrations of LPS for 24 h. Bars indicate means ± SEM. *$p < 0.05$, **$p < 0.01$, ***$p<0.001$.

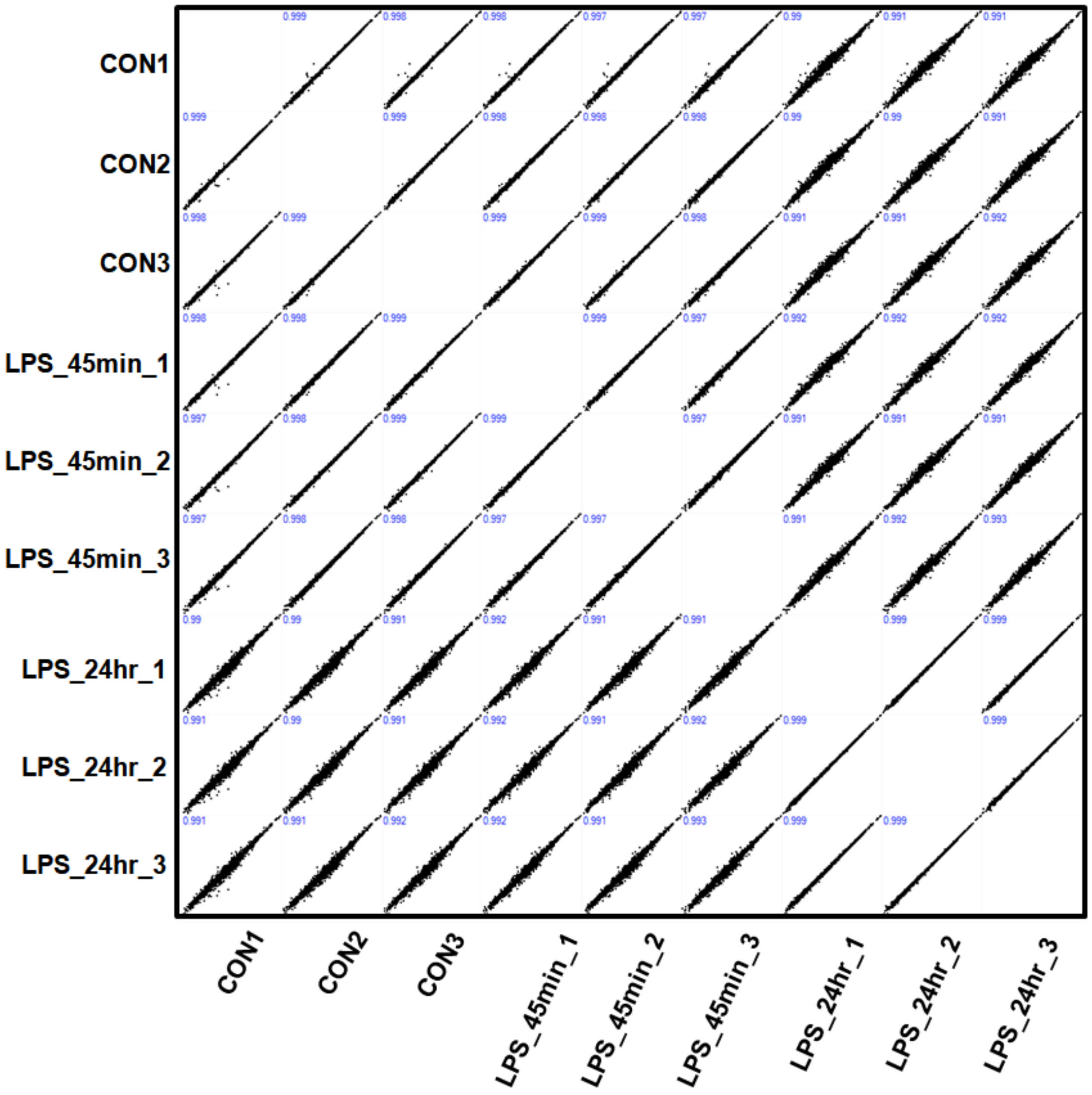


**Figure S2.** Reproducibility of global proteomic quantification. Multi-scatter plot of label-free protein intensities between different replicates of the samples was depicted with the Pearson correlation coefficient values.

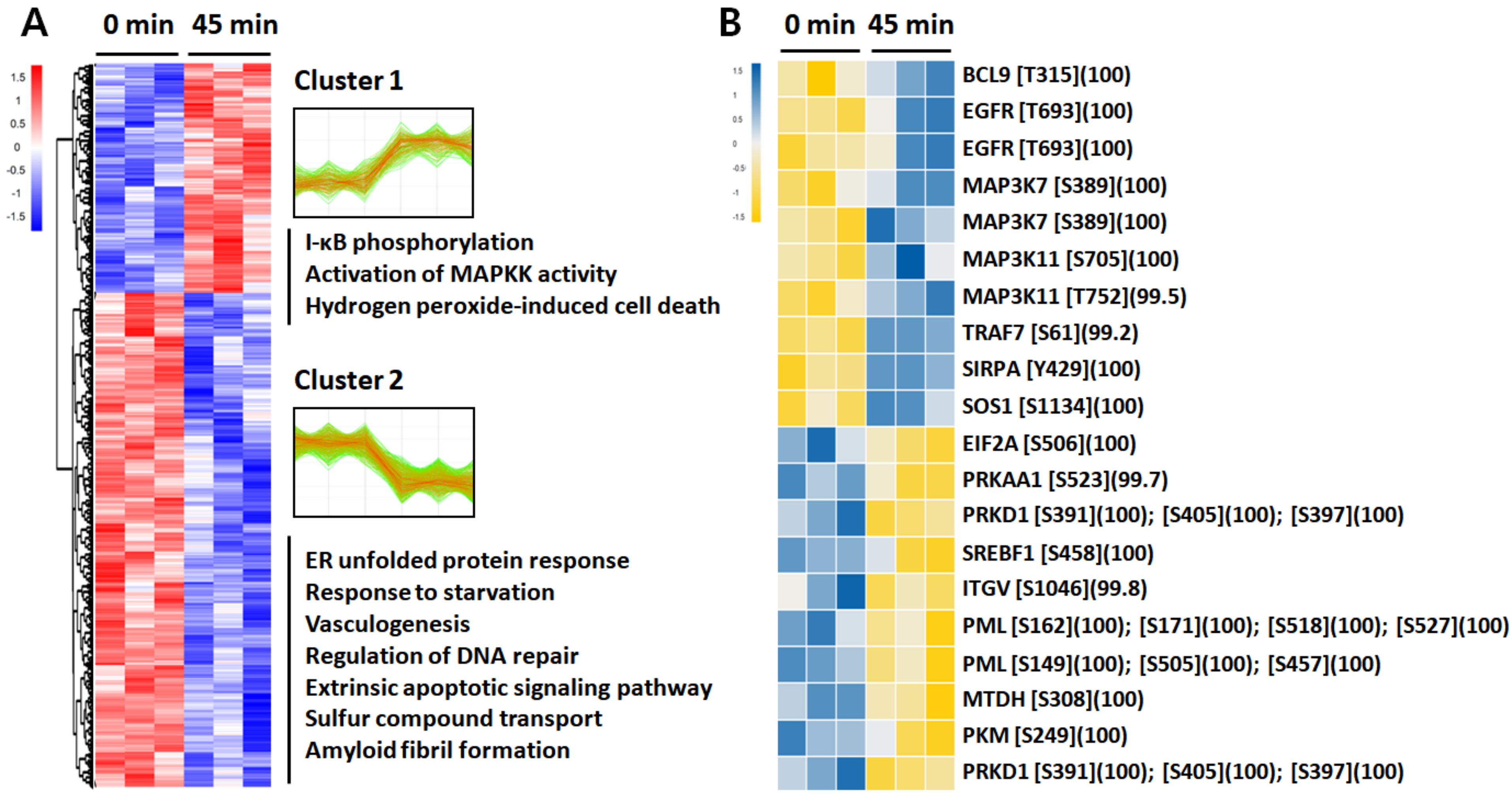


**Figure S3.** Hierarchical clustering of the identified phosphoproteins with differential phosphosite intensities. (A) Hierarchical cluster analysis of significantly altered phosphosites upon 45 min treatment of 50 µg/ml LPS, according to their abundance profile with significantly enriched biological process gene ontology terms within clusters. (B) Heat map showing phosphorylation status of 16 phosphosites that were significantly changed between two time point measurement.

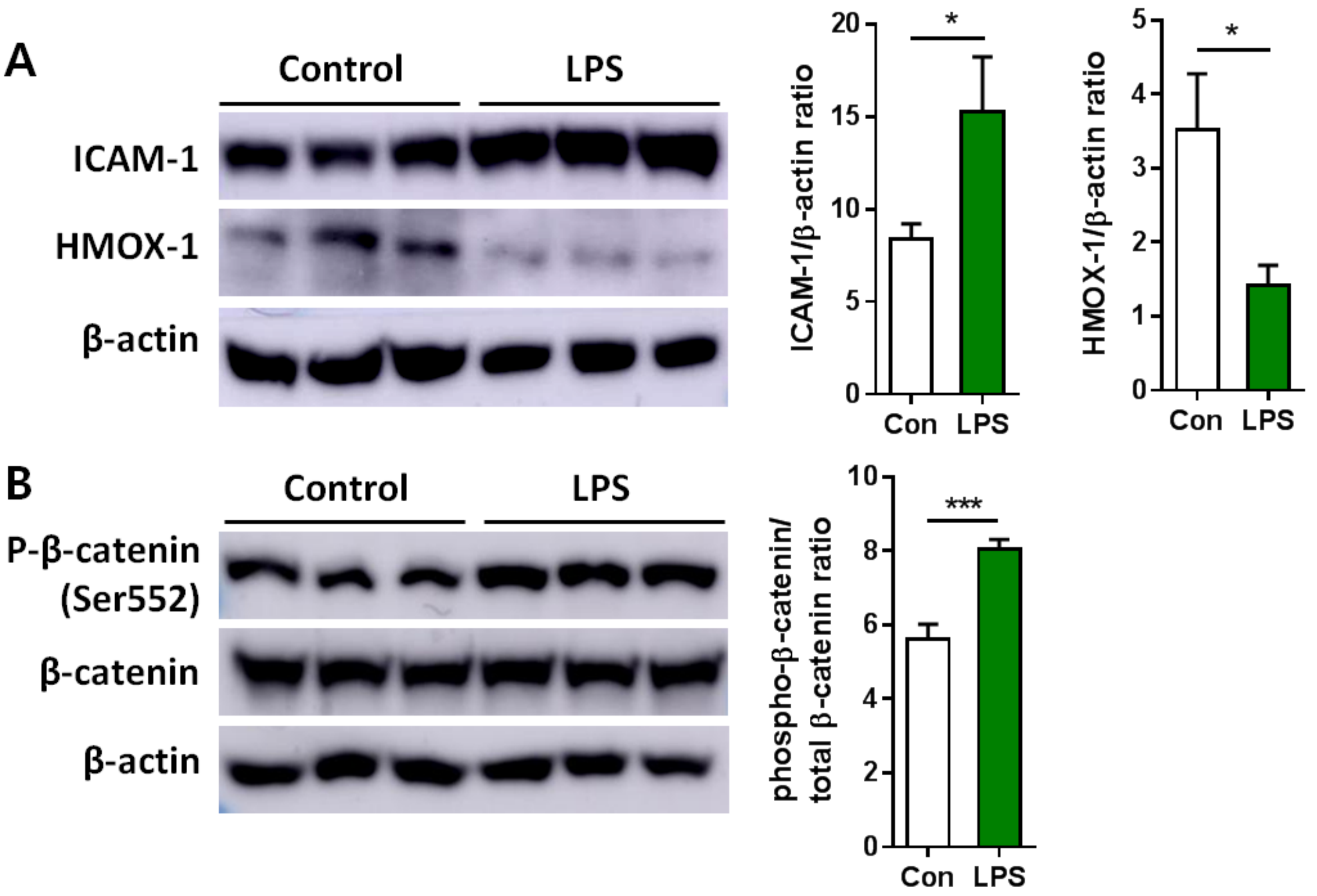


**Figure S4.** Western blot analysis of proteins and phosphoprotein included in the pathways related to inflammation-driven pathogenesis in ARPE-19 cells. ARPE-19 cells were challenged with 50 μg/ml LPS for 24 h. (A) Representative western blot protein bands and histograms for ICAM-1 and HMOX-1 normalized to β-actin. (B) Representative western blot protein bands and histograms of phospho-β-catenin (Ser552) normalized to total form. Bars indicate means ± SEM. *$p < 0.05$, ***$p < 0.001$.
